# Caribbean Women Face Higher Obesity and Diabetes Amid Socioeconomic Struggles

**DOI:** 10.7759/cureus.91259

**Published:** 2025-08-29

**Authors:** Cesar Barrabi, Amanda Fowler

**Affiliations:** 1 Epidemiology and Public Health, The Chicago School, Chicago, USA; 2 Medical Education, Western Atlantic University School of Medicine, Freeport, BHS

**Keywords:** caribbean health disparities, diabetes prevalence, food insecurity and health, gender disparities, healthcare access barriers, noncommunicable diseases, obesity epidemiology, public health inequities, socioeconomic determinants of health, women's health outcomes

## Abstract

Background

Women in the Caribbean region experience significant health disparities shaped by intersecting medical and socioeconomic challenges. High rates of obesity, diabetes, and maternal mortality have been observed alongside persistent inequalities in development, employment, and food access. This study aimed to assess gender-based disparities in obesity, diabetes, reproductive health, and socioeconomic conditions across Caribbean countries, comparing outcomes to North America to identify structural drivers of women’s health inequities.

Methods

We analyzed regional and gender-based trends in health and social outcomes across up to 30 Caribbean countries. Publicly available data from 2019 to 2022 were compiled to assess noncommunicable disease prevalence, reproductive health indicators, and key economic metrics. Caribbean nations were compared to the United States and Canada to contextualize findings.

Results

Women in the Caribbean had higher obesity prevalence and a greater proportion of diabetes-related deaths compared to men. The region also reported elevated maternal and infant mortality, lower inequality-adjusted development scores, and wider gender gaps in unemployment. Food insecurity affected more than 40% of the population in several countries. Adolescent fertility and mortality rates were also higher in the Caribbean than in North America. Multivariate analyses revealed strong associations between chronic disease outcomes and structural indicators such as healthcare access and economic inequality.

Conclusion

Caribbean women face compounding health risks driven by overlapping medical, economic, and social vulnerabilities. These disparities highlight the need for coordinated regional strategies that go beyond behavioral health to address the broader structural determinants of health and gender equity.

## Introduction

Gender health disparities are a well-documented global issue, with women often facing worse outcomes in chronic diseases, mental health, and access to healthcare [1]. In the Caribbean, these disparities are particularly stark, with women facing higher rates of obesity and diabetes and poorer reproductive health outcomes, as demonstrated in regional mortality assessments and global risk analyses [2,3]. While high-income countries like the United States of America (US) and Canada (CAN) also struggle with noncommunicable diseases (NCDs), Caribbean nations such as the Bahamas and Barbados experience these challenges within more fragile healthcare systems and greater socioeconomic inequalities [4]. Although lifestyle factors like poor nutrition and physical inactivity are widely recognized contributors [5], the deeper socio-economic influences on NCDs among Caribbean women remain underexplored.

Despite its relative wealth, the Bahamas exhibits high obesity rates and diabetes-related mortality, consistent with global trends showing disproportionate NCD burdens in middle-income nations [6]. Caribbean women have 65% higher odds of diabetes and over three times the odds of obesity compared to men [4]. These disparities highlight the need for targeted policy interventions that expand healthcare access, address food insecurity, and reduce socioeconomic inequality, particularly among young adults and women [7]. However, while these issues are well-documented in North America, research that explores their specific impact on Caribbean women remains limited.

Beyond chronic diseases, reproductive health poses additional risks. In the United States [8], maternal mortality remains high due to inadequate chronic disease care and poor postpartum follow-up, while in Latin America and the Caribbean, persistent structural inequities continue to drive wide disparities in maternal health outcomes [9]. Yet much of the literature focuses on Latin America, leaving a critical gap in understanding maternal health challenges unique to the non-Latin Caribbean. Additionally, Caribbean women face higher maternal mortality rates, early menstruation, and late pregnancies, which contribute to breast cancer risks and poor reproductive outcomes [9,10]. The stigma around conditions like cervical cancer further limits healthcare access, creating a cycle of late diagnoses and higher mortality [11]. These observed disparities suggest that comprehensive approaches integrating medical treatment and addressing socioeconomic barriers may be beneficial, consistent with findings from prior studies.

We aimed to examine gender-based disparities in obesity and diabetes and their association with structural socioeconomic variables, using data from up to 30 non-Latin Caribbean countries. This study contributes new regional evidence on how structural inequities, including healthcare access, socioeconomic vulnerability, and gender-specific risks, shape women’s health across the Caribbean. By integrating chronic disease, reproductive health, and policy data, our findings provide a foundation for understanding the multifaceted drivers of health inequality in the region. This article was previously posted as a preprint on medRxiv on March 22, 2025 (https://doi.org/10.1101/2025.03.22.25324462).

## Materials and methods

Study design and data collection

This cross-sectional study analyzed gender disparities in health outcomes across up to 30 Non-Latin Caribbean and reference countries using publicly available data from the Pan American Health Organization (PAHO) for most health indicators, the United Nations Development Programme (UNDP) for Human Development Index (HDI), Inequality-Adjusted HDI, and selected economic metrics, and the International Diabetes Federation (IDF) for diabetes prevalence and hyperglycemia in pregnancy rates by region. The analysis included metrics on noncommunicable diseases (NCDs), reproductive health outcomes, and socioeconomic indicators, comparing these to benchmarks in North America (NA). All data used in this study were aggregate, country-level indicators obtained from public sources. No individual-level or patient-specific data were accessed or analyzed. Most health and socioeconomic indicators used in this study were from 2019, the most recent year with comprehensive and comparable country-level data. These data sources were selected for their coverage, credibility, and relevance, particularly given the limited availability of peer-reviewed literature addressing Caribbean health outcomes. Analyses were performed using the maximum number of countries available for each indicator while retaining all countries to preserve regional representation. Sixteen countries contributed data for at least 85% of variables; the remainder had more limited coverage. A table of the data used for the study is included in the Appendices section.

The primary outcomes assessed were obesity and diabetes prevalence, maternal and adolescent health metrics, and socioeconomic disparities, including Human Development Index (HDI) scores, food insecurity, and unemployment rates. Several key health indicators, particularly mortality outcomes, required clarification due to differences in how they were defined or reported across primary sources. For transparency, we explain how select metrics were interpreted or calculated in this analysis. External mortality was classified as deaths from injuries, violence, traffic incidents, and other unintentional causes, following PAHO definitions. NCD mortality included deaths from chronic conditions such as cardiovascular disease, cancer, diabetes, and chronic respiratory illness. Diabetes-related mortality was assessed using two complementary measures. The first was the age-adjusted diabetes mortality rate, defined by PAHO as the number of deaths per 100,000 population in which diabetes was identified as the underlying cause of death. This determination was based on national death certificate data coded using the International Classification of Diseases, submitted annually by member states to PAHO. This metric was used to compare overall diabetes mortality across regions. The second measure was the proportion of deaths due to diabetes, calculated by dividing the diabetes mortality rate by the general mortality rate (both age-adjusted) for each group. This proportion allowed us to assess the relative contribution of diabetes to total mortality within each gender group in the Caribbean. Additional variables related to lifestyle, healthcare access, and reproductive health were included to provide a broader context.

In a separate analysis, we assessed national-level policy implementation related to NCD prevention using data from the 2022 PAHO ENLACE interactive scorecard. This tool evaluates country-level progress on commitments outlined in the 2011 UN Political Declaration and the 2014 UN Outcome Document on NCDs. Four policy indicators were selected: (1) national evidence-based clinical guidelines for NCD management, (2) policies or campaigns promoting physical activity, (3) implementation of the International Code of Marketing of Breast-milk Substitutes, and (4) restrictions on the marketing of unhealthy foods and beverages to children. Each indicator was classified by PAHO as fully implemented (green), partially implemented (yellow), not implemented (red), or unknown (grey).

For the policy implementation analysis, we used 2022 ENLACE data, as no comparable implementation metrics were collected in 2019, and earlier rounds used different definitions. Countries were grouped into two regions: North America (n = 2; Canada and the United States) and the Caribbean (n = 13; Antigua and Barbuda, Bahamas, Barbados, Belize, Dominica, Grenada, Guyana, Jamaica, Saint Kitts and Nevis, Saint Lucia, Saint Vincent and the Grenadines, Suriname, and Trinidad and Tobago).

Data analysis

All data processing and visualization, including scatterplots and trend lines, were conducted using Microsoft Excel. More advanced statistical analyses, such as Pearson Correlations and Principal Component Analysis (PCA), were performed in Python, as described later. For gender-wise comparisons, Student’s t-tests were used to compare male-to-female differences within Caribbean nations for each metric. Welch’s t-tests were used for country-level comparisons, grouping the United States and Canada as one group (North America or NA) and up to 30 non-Latin Caribbean nations as the other group (Caribbean or CAR), with a significance cutoff of p < 0.05 [12]. This test was chosen to account for unequal sample sizes. Data for gestational diabetes rates for the Caribbean were retrieved from the International Diabetes Federation (IDF, 2021), using regional data. A Geographic Information System (GIS) map of the Caribbean was generated in Quantum Geographic Information System (QGIS) 3.34 T using Database of Global Administrative Areas (GADM) shapefiles, with mapping performed by Fiverr professional Ayesha Suraweera. The base map shapefiles were obtained from the Database of Global Administrative Areas (GADM), available at https://gadm.org/data.html, and used under a Creative Commons Attribution (CC-BY)-compatible license. No statistics were performed in QGIS; this is simply reporting values obtained from IDF.

Due to incomplete reporting across key indicators in several countries, data imputation was performed to allow their inclusion in correlation and PCA analyses while minimizing bias from missingness. Countries with less than 75% of data for each variable were excluded, and the remaining missing data for 16 countries were imputed using Predictive Mean Matching within the Multiple Imputation by Chained Equations framework in Python. This approach maintains the original data distribution by matching predicted values to the closest observed values, ensuring realistic imputation. After data imputation, Pearson correlation coefficients were calculated to examine associations.

PCA was performed using the imputed data to reduce the dimensionality of the dataset with socio-economic and health indicators across the 16 countries. Variables with high collinearity were removed based on Pearson correlations before PCA, and additional variables were hand-selected based on relevance to women’s health metrics, socio-economic indicators, and other health-related metrics. In this analysis, normality was assessed for all 51 variables using the Shapiro-Wilk test, with 76% meeting normality criteria, which was deemed sufficient to proceed with PCA. A scree plot confirmed that the first two principal components explained most of the variance. Ward’s hierarchical clustering was applied to the PCA scores to group countries based on their socio-economic and health profiles, while the elbow method was applied to select the number of clusters. Bootstrapping was performed with 1,000 iterations to assess the stability of the PCA results, achieving a 95% confidence interval.

Ethical considerations

This study used publicly available, aggregated secondary data from international organizations and did not involve human subjects. Therefore, formal ethical approval was not required.

## Results

The Caribbean nations show distinct gendered mortality patterns, with women facing higher diabetes mortality

**Figure 1:**
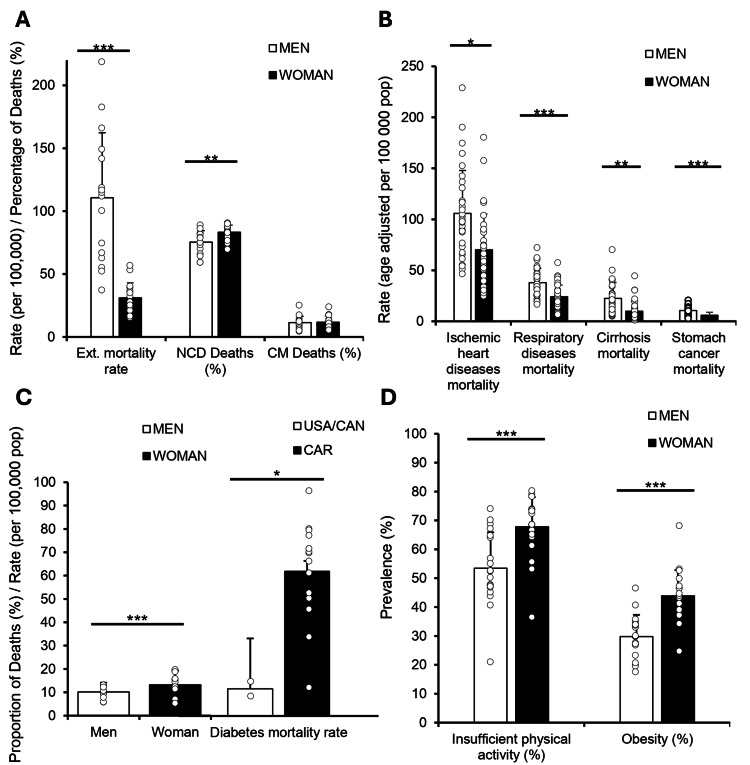
Gender-based disparities in mortality and health indicators across the Caribbean region All data were obtained from PAHO using 2019 data. (A) A Student's t-test was performed examining the external causes mortality rate (Ext. Mortality rate, age-adjusted per 100,000 population) and the proportion of deaths from noncommunicable diseases (NCDs) and communicable diseases (CDs) (%) by gender. (B) A Student's t-test was performed measuring the age-adjusted mortality rates (per 100,000 population) for ischemic heart disease, respiratory diseases, cirrhosis, and stomach cancer by gender. (C) A Student's t-test was performed to measure the diabetes mortality rate (per 100,000 population) and a Welch’s t-test was used to compare proportional diabetes mortality (%) between the USA/Canada and the Caribbean nations by gender. (D) Prevalence of insufficient physical activity and obesity (%) by gender. Asterisks denote statistical significance: p < 0.05 = *, p < 0.01 = **, p < 0.001 = **.

We first examined 2019 PAHO data across 30 Caribbean nations to evaluate gender-specific health outcomes. External mortality rates were significantly higher in men (110.68 per 100,000) compared to women (31.01; p < 0.001), while NCD mortality was higher in women (83.18%) than in men (75.33%; p < 0.01). Communicable Disease (CD) mortality showed no significant gender difference (Figure 1A). To explore which conditions contributed to the observed gender disparity in NCDs, we identified the four NCD categories with the largest and most consistent male excess in mortality across countries: ischemic heart disease, respiratory diseases, cirrhosis, and stomach cancer (all p < 0.05; Figure 1B). These conditions were selected to illustrate that, outside of diabetes and obesity, men generally experience higher NCD mortality than women in the Caribbean. We next examined diabetes-related mortality, given its known burden in the region. Women had a higher proportion of diabetes-related deaths than men (10.58% vs. 7.23%; p < 0.01). When comparing regions, the age-adjusted diabetes mortality rate was substantially higher in the Caribbean nations than in North America (62.22 vs. 11.65 deaths per 100,000 population; p < 0.001), highlighting a striking regional disparity in the burden of diabetes-related deaths (Figure 1C).

To assess potential contributors to this pattern, we analyzed lifestyle metrics. Women in the Caribbean reported significantly higher rates of insufficient physical activity compared to men (67.79% vs. 53.47%; p < 0.001), and obesity prevalence was also higher among women (43.88% vs. 29.77%; p < 0.001; Figure 1D).

The Caribbean region faces greater socioeconomic and health challenges compared to the USA/Canada

**Figure 2:**
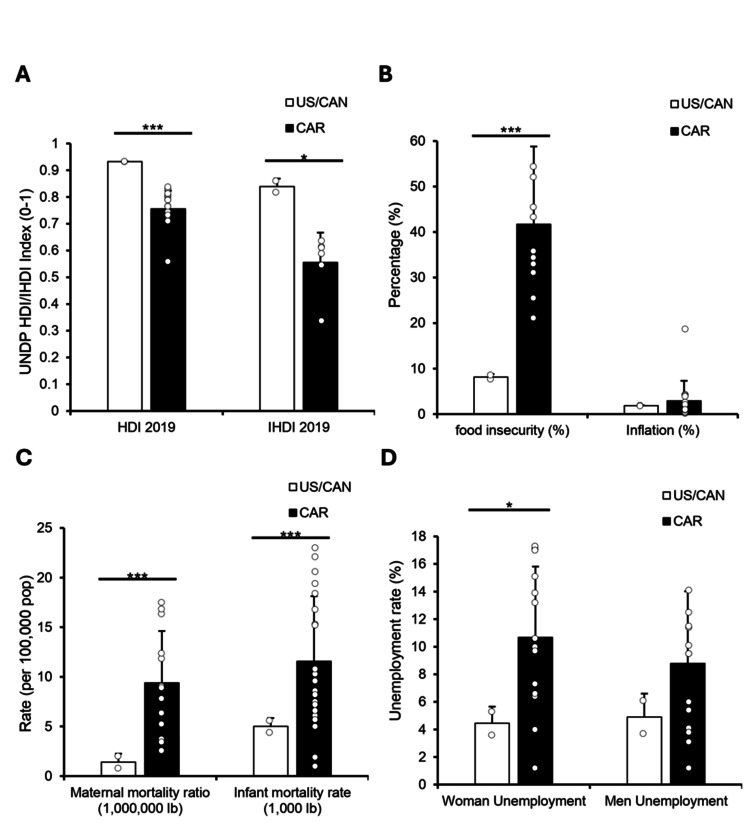
The Caribbean region faces greater socioeconomic and health challenges compared to the USA/Canada Human Development Index (HDI) and Inequality-adjusted HDI (IHDI) data were obtained from the United Nations Development Programme (UNDP) for 2019, while all other data were from the Pan American Health Organization (PAHO) for 2019. Welch’s t-tests were used to compare the USA/Canada (US/CAN) with 30 Caribbean nations across all variables. (A) HDI and IHDI scores comparing the US/CAN and Caribbean regions. (B) Food insecurity prevalence (%) and inflation (%) in the US/CAN and Caribbean regions. (C) Homicide and suicide mortality rates (per 100,000 population) by region. (D) Unemployment rates (%) for men and women in the US/CAN and Caribbean regions. Asterisks denote statistical significance: p < 0.05 = *, p < 0.01 = **, p < 0.001 = **.

We then aimed to expand on the observed gendered health disparities and assessed socioeconomic conditions in the Caribbean relative to North America (NA). The region had significantly lower Human Development Index (HDI) scores (0.76 vs. 0.93; p < 0.001) and Inequality-Adjusted HDI (0.56 vs. 0.84; p < 0.01), reflecting more limited development and deeper structural inequality (Figure 2A). Food insecurity was also considerably higher in the Caribbean (41.71%) compared to NA (8.15%; p < 0.001), despite no significant regional differences in overall inflation rates. Rates in Haiti (18.7), Aruba (4.3), and Barbados (4.1) were the highest recorded, suggesting that inflation alone does not explain the region’s more severe access challenges (Figure 2B). We next explored mortality from external causes. Homicide mortality was significantly higher in the Caribbean (7.31) than in NA (1.55; p < 0.01), while suicide mortality showed no significant difference (6.1 vs. 3.87), though elevated rates in Guyana (17.0) and Suriname (11.8) contributed to regional variability (Figure 2C). To examine economic instability through a gendered lens, we compared unemployment rates. Women's unemployment was significantly higher in the Caribbean (10.67%) than in NA (4.45%; p < 0.01), whereas men’s unemployment showed no regional difference (Figure 2D). 

Reproductive and early-life health indicators underscore deep regional and gender inequities

**Figure 3:**
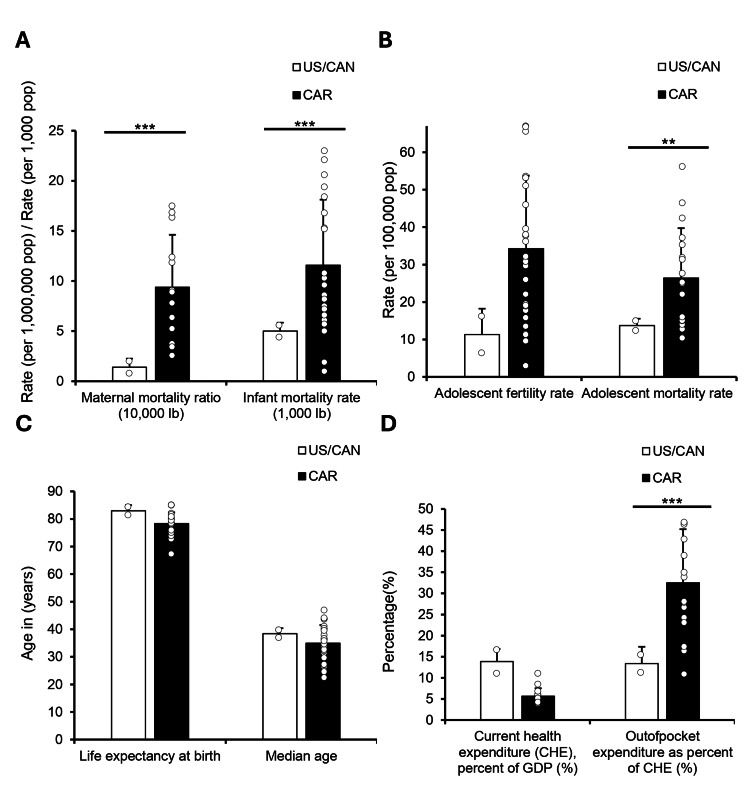
The Caribbean nations exhibit greater health and economic vulnerabilities compared to the USA/Canada Welch’s t-tests were used to compare the USA/Canada (US/CAN) with 30 Caribbean nations across all variables. (A) Maternal mortality ratio (per 1,000,000 live births) and infant mortality rate (per 1,000 live births) comparing the US/CAN and Caribbean regions. (B) Adolescent fertility rate (per 1,000 population) and adolescent mortality rate (per 100,000 population) by region. (C) Life expectancy at birth and median age (in years) for the US/CAN and CAR regions. (D) Current health expenditure as a percentage of Gross Domestic Product (GDP) and out-of-pocket expenditure as a percentage of current health expenditure (%) comparing the US/CAN and Caribbean regions. Asterisks denote statistical significance: p < 0.05 = *, p < 0.01 = **, p < 0.001 = **.

To build on the observed health and socioeconomic disparities, we examined reproductive and early-life health outcomes in the Caribbean compared to NA. Maternal mortality was significantly higher in the Caribbean (9.39 per 10,000 live births) than in NA (1.41; p < 0.001), and infant mortality showed a similar trend, with rates more than twice as high in the Caribbean (11.58 vs. 5 per 1,000 live births; p < 0.001; Figure 3A). We next assessed adolescent health metrics to identify early-life contributors to regional disparities. Adolescent fertility rates were higher in the Caribbean region (34.26 per 10,000) and trended toward significance when compared to NA (11.3; p = 0.05006). Adolescent mortality was significantly elevated in the Caribbean region (26.38 vs. 13.7 per 10,000; p < 0.01), highlighting additional risk in this age group (Figure 3B). To contextualize these disparities, we examined broader demographic and health system indicators. Life expectancy, median age, and total health spending did not differ significantly between regions. However, out-of-pocket health expenditures were more than twice as high in the Caribbean (32.5%) relative to NA (13.4%; p < 0.01), indicating a heavier financial burden on individuals seeking care (Figures 3C-3D).

**Figure 4:**
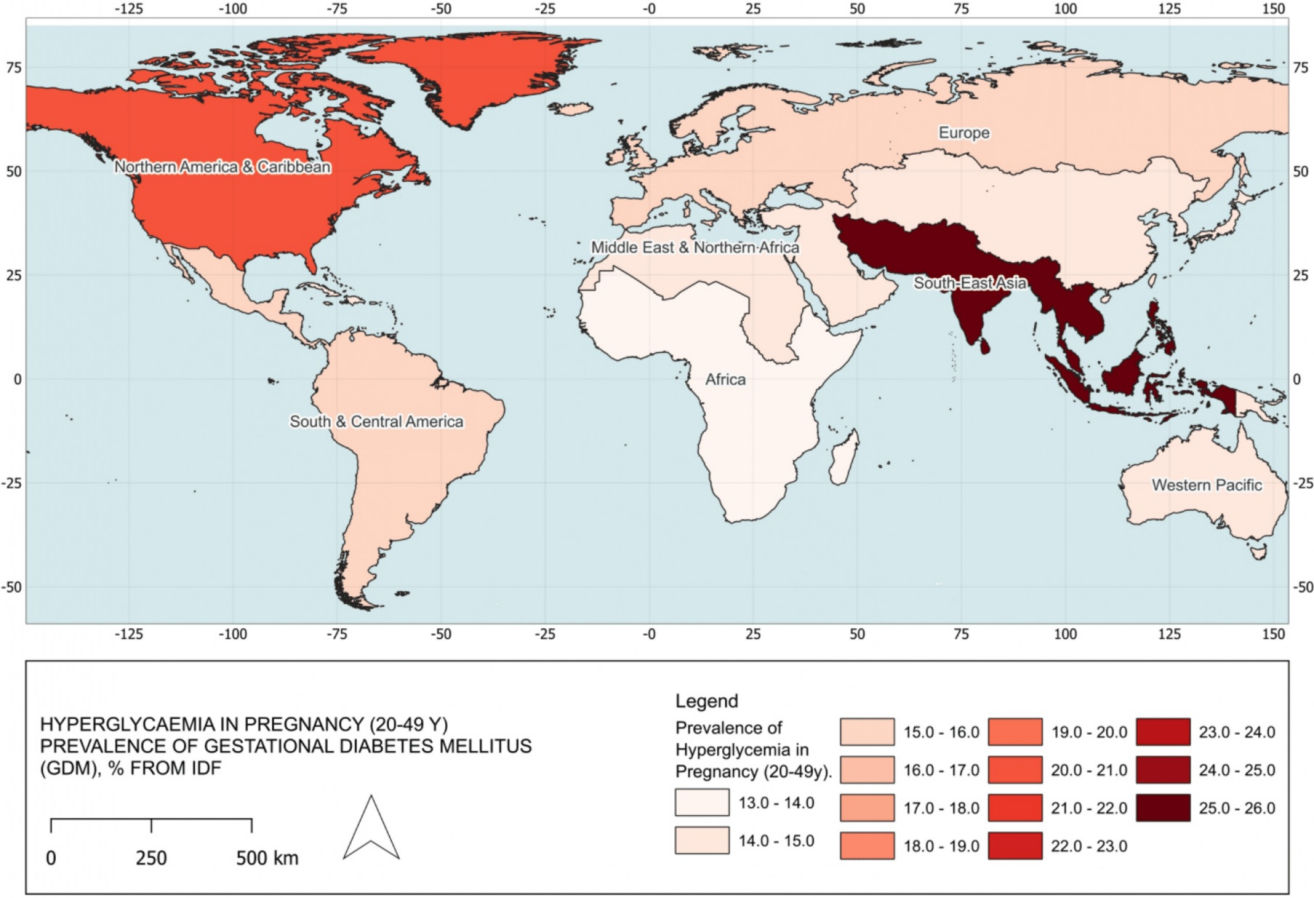
Prevalence of gestational diabetes mellitus (GDM) by region, 2021 All data were obtained from the International Diabetes Federation (IDF) for the year 2021. The dataset represents the prevalence of hyperglycemia in pregnancy (age 20-49) across different regions. The map was created in QGIS 3.34 T using shapefiles from the Database of Global Administrative Areas (GADM), available at https://gadm.org/data.html. Darker shades indicate higher prevalence rates, highlighting disparities in gestational diabetes across the regions. South-East Asia: 25.9%, North America and Caribbean: 20.7%, South and Central America: 15.8%, Europe: 15%, Middle East and North Africa: 14.1%, Western Pacific: 14%, Africa: 13%.

We also explored gestational diabetes mellitus (GDM) prevalence as a marker of maternal health vulnerability. Both the Caribbean and North America reported a GDM prevalence of 20.7%, placing the region behind South-East Asia (25.9%) but ahead of South and Central America (15.8%; Figure 4). These rates signal an emerging challenge for maternal health across the hemisphere.

Diabetes and obesity outcomes strongly correlate with socioeconomic development and healthcare access in Caribbean populations

**Figure 5:**
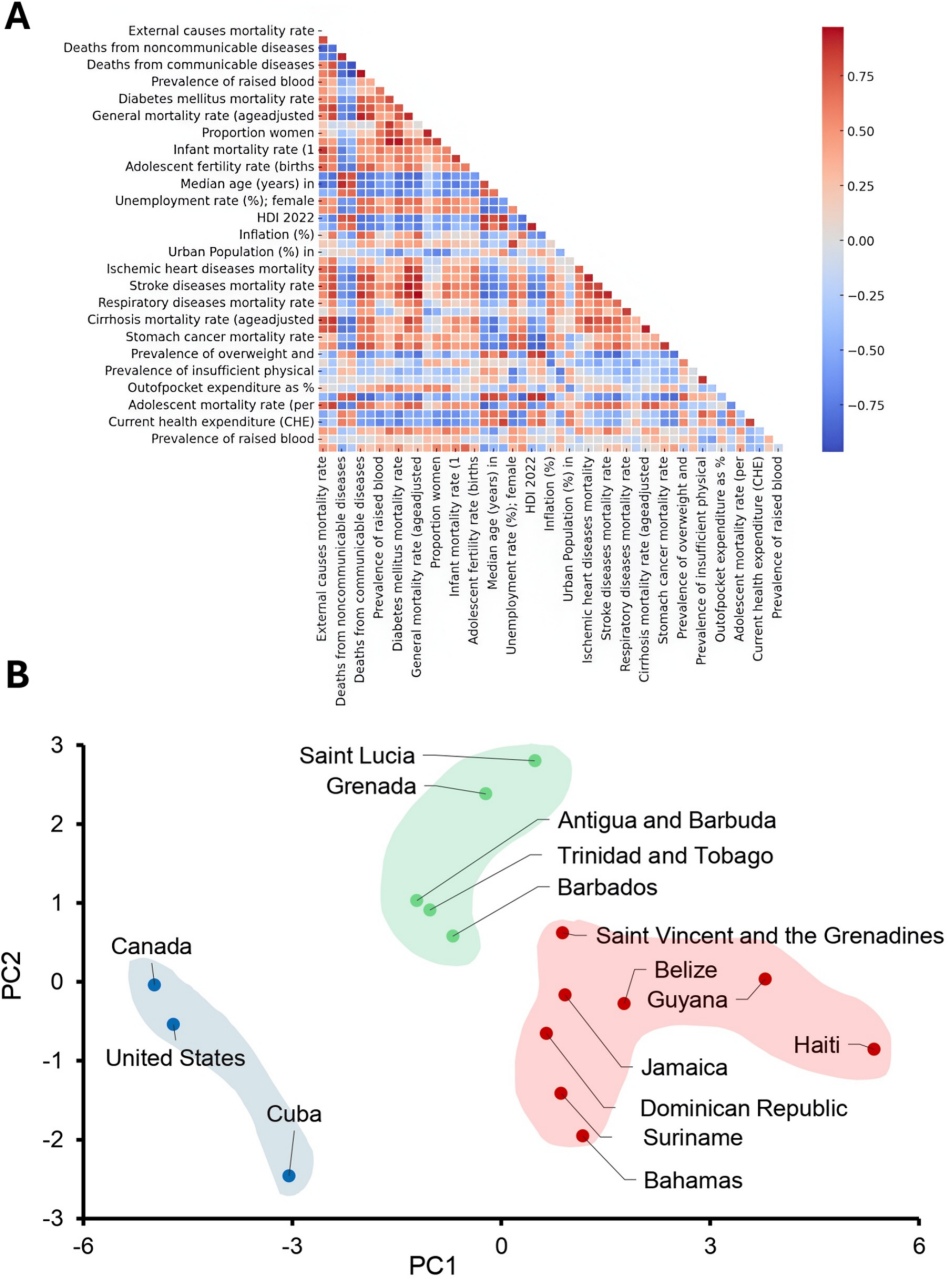
Correlation of health and demographic variables and distinct regional health profiles in the Caribbean Only data with at least 75% availability were included for Pearson correlation and Principal Component Analysis (PCA). (A) illustrates the relationships between health and demographic variables across countries with at least 75% data availability. Positive correlations are shown in red, and negative correlations in blue, with stronger correlations indicated by more intense colors. (B) PCA identified three distinct clusters of countries. Principal Component 1 (PC1) reflects socioeconomic status and health outcomes, while Principal Component 2 (PC2) represents healthcare access and lifestyle-related health risks. Clusters highlight the USA, Canada, and Cuba with favorable health outcomes, intermediate Caribbean nations with moderate health and socioeconomic challenges, and more vulnerable Caribbean nations with higher adolescent mortality and fertility rates.

We examined how socioeconomic conditions relate to population health by conducting Pearson correlation analyses across Caribbean nations with at least 75% data availability, with results visualized in a correlation heatmap (Figure 5A). Missing values were imputed specifically for these analyses. Diabetes mortality showed a strong inverse correlation with inequality-adjusted development, with higher IHDI values associated with lower age-adjusted diabetes deaths (r = -0.884). Adult obesity prevalence was also negatively associated with healthcare system strength, as measured by the Universal Health Coverage (UHC) index (r = -0.841). These findings align with previous reports demonstrating that countries with lower HDI tend to have higher diabetes-related mortality.

Economic development was moderately correlated with multiple reproductive and adolescent health outcomes. Higher GDP per capita was associated with increased low birth weight prevalence (r = 0.610) and lower adolescent fertility (r = -0.710), suggesting complex links between economic status, maternal health, and early-life reproductive patterns [13]. Finally, adolescent mortality (per 1,000 females aged 15-19) showed a moderate positive correlation with preterm birth rates (r = 0.583), indicating that adverse birth outcomes may contribute to elevated mortality in this age group [14]. These findings point to the layered relationship between development, healthcare systems, and health outcomes in Caribbean populations, particularly among women and adolescents.

To identify underlying patterns in health and socioeconomic indicators, we performed Principal Component Analysis (PCA) across countries with sufficient data availability. Two principal components explained the majority of variance in the dataset (Figure 5B). PC1 accounted for 49.0% of the variance and reflected a gradient from stronger socioeconomic conditions and lower NCD mortality to higher adolescent fertility and mortality. Positive contributors included adolescent fertility and adolescent mortality, while negative contributors included GDP per capita, inequality-adjusted HDI, and female NCD mortality. PC2 explained an additional 13.2% of the variance and captured variation in healthcare access and lifestyle-related factors, driven by out-of-pocket expenditure and insufficient physical activity.

Hierarchical clustering applied to PCA scores revealed three country groups with distinct profiles. The first cluster, Canada, the United States, and Cuba had the most favorable outcomes, including the lowest adolescent mortality (1.41 per 1,000) and fertility rates (24.6 births per 1,000), and the highest life expectancy (81.9 years). The second cluster, Antigua and Barbuda, Barbados, Grenada, Saint Lucia, and Trinidad and Tobago, showed intermediate outcomes, with adolescent mortality of 1.74 per 1,000, adolescent fertility of 35.6 per 1,000, and a life expectancy of 77.7 years.

The third cluster, composed of the Bahamas, Belize, Dominican Republic, Guyana, Haiti, Jamaica, Saint Vincent and the Grenadines, and Suriname, demonstrated the greatest vulnerability. This group had the highest adolescent mortality (3.70 per 1,000), adolescent fertility (54.0 per 1,000), and the lowest life expectancy (73.7 years). It also reported the highest maternal mortality (123.1 per 100,000), with Haiti as an outlier at 128.4 per 100,000.

Caribbean and North American countries differ in policy implementation for NCD prevention

**Figure 6:**
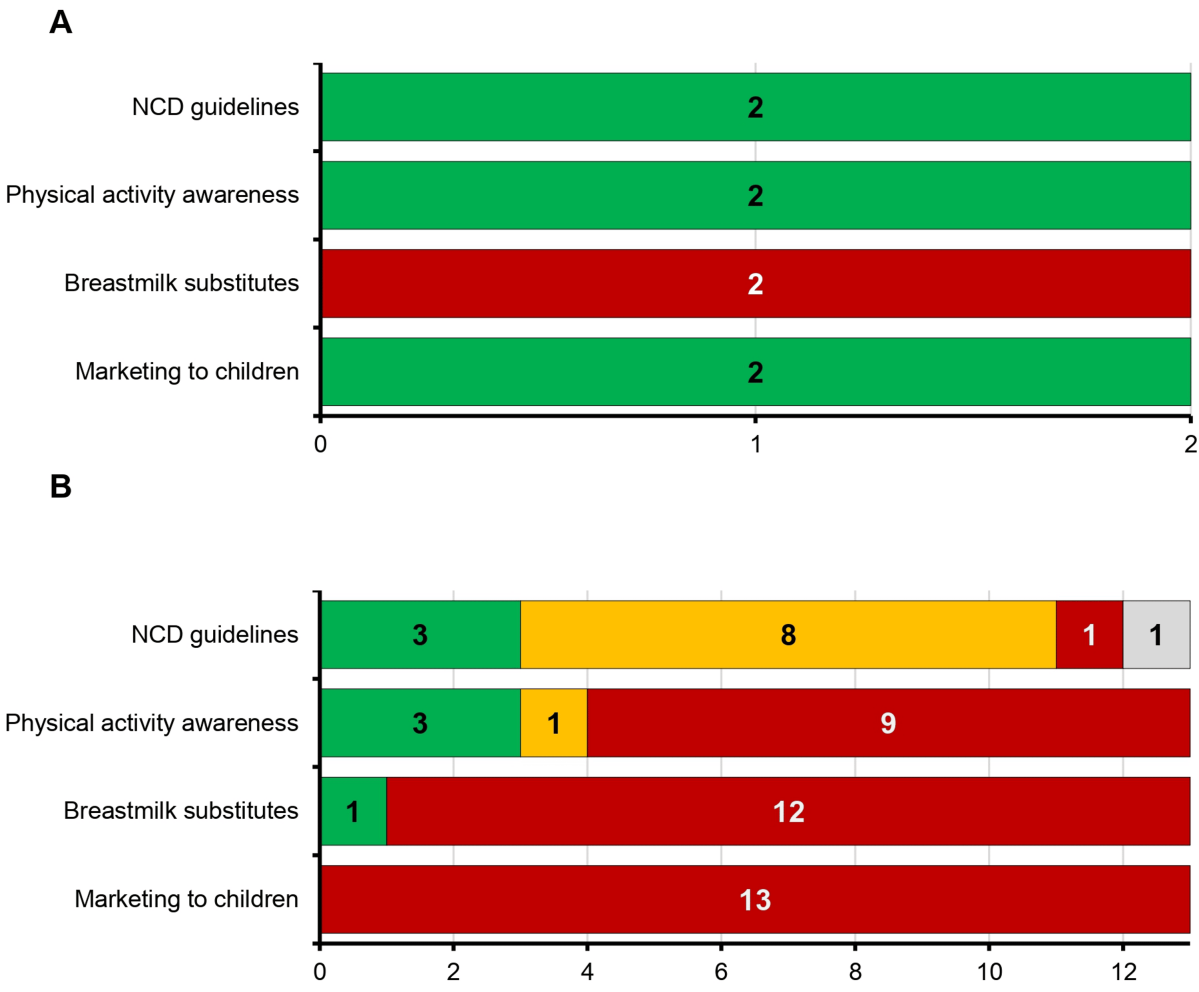
Implementation of NCD-related policies across the Caribbean region and North America Data are from the 2022 Pan American Health Organization (PAHO) ENLACE interactive non-communicable disease (NCD) scorecard. Each bar represents the number of countries classified by implementation status across four key policy areas: national NCD guidelines, physical activity awareness, regulation of breastmilk substitutes, and marketing restrictions to children. Green indicates full implementation, yellow partial implementation, red no implementation, and grey unknown status. Panel A shows results for North America (n = 2; United States and Canada), both of which fully implemented three of the four policies, with no action taken on breastmilk substitute regulation. Panel B displays results for the Caribbean (n = 13; Antigua and Barbuda, Bahamas, Barbados, Belize, Dominica, Grenada, Guyana, Jamaica, Saint Kitts and Nevis, Saint Lucia, Saint Vincent and the Grenadines, Suriname, and Trinidad and Tobago), where implementation was more variable. Most Caribbean countries had not implemented policies on marketing to children or breastmilk substitutes, while a subset had made progress on NCD guidelines and physical activity awareness.

National action on NCD prevention varies widely across the Americas, as shown by comparisons of four key policy indicators from the 2022 PAHO ENLACE NCD scorecard. This analysis included North America (NA: United States and Canada) and 13 countries in the Caribbean region (CAR: Antigua and Barbuda, Bahamas, Barbados, Belize, Dominica, Grenada, Guyana, Jamaica, Saint Kitts and Nevis, Saint Lucia, Saint Vincent and the Grenadines, Suriname, and Trinidad and Tobago) (Figure 6).

We began by examining the presence of national NCD guidelines, which were fully implemented in both NA countries. In the Caribbean region, full implementation was reported in Saint Lucia, Saint Vincent and the Grenadines, and Trinidad and Tobago. Six countries, including Belize, Barbados, Bahamas, Guyana, Jamaica, and Suriname, had partially implemented guidelines, while the remaining four had taken no action. This mixed picture suggests that although some Caribbean countries are progressing toward national NCD care standards, full adoption remains limited.

We then assessed physical activity awareness policies, which were fully implemented in both NA countries. In CAR, Barbados, Belize, and Saint Lucia had achieved full implementation, while Suriname and Trinidad and Tobago reported partial implementation. The remaining eight countries, including Jamaica, the Bahamas, and Antigua and Barbuda, had no such policies in place. The gap in physical activity promotion across much of the region may reflect both resource limitations and the absence of coordinated national strategies.

Regulation of breastmilk substitutes showed minimal uptake in both regions. Neither NA country had implemented policies aligned with the International Code of Marketing of Breast-milk Substitutes. Among Caribbean countries, only Guyana had fully implemented this policy. All others, including Saint Lucia, Jamaica, and Saint Vincent and the Grenadines, had not adopted any regulatory measures. Despite growing global consensus on the importance of protecting breastfeeding, this remains a neglected policy area across most of the Caribbean.

Finally, marketing restrictions on children revealed the sharpest contrast. Canada and the United States had both fully implemented these protections. In contrast, all 13 CAR countries reported no implementation. This includes countries like Barbados and Belize that have otherwise made progress in other areas. The complete lack of child-targeted marketing regulation across the Caribbean raises concerns about commercial influences on childhood diet and long-term NCD risk.

## Discussion

This study examined gender disparities in health outcomes across the Caribbean, focusing on how socioeconomic conditions, healthcare access, and systemic inequalities disproportionately affect women's health. The analysis highlighted the intersection of chronic diseases, reproductive health disparities, and economic stressors, with a particular focus on the Bahamas due to its notable obesity rates, limited healthcare access, and elevated mortality metrics. Building upon prior research, this study provides novel insights into the complex relationship between gender, health outcomes, and socioeconomic factors in the region [2,3].

Our findings revealed significant gender disparities in NCDs, including obesity and diabetes. Women in the Bahamas exhibited higher obesity rates (54.1%) than men (38.3%), a disparity that aligns with broader NCD trends observed across the Caribbean [2,3]. Previous research has shown that socioeconomic inequalities contribute to adverse health outcomes in the region, including elevated risks for preterm birth and other preventable conditions [15]. These systemic inequities often interact with healthcare access challenges, including high out-of-pocket costs and limited preventive services, which delay diagnosis and treatment for chronic diseases [15,16]. Our Pearson correlation findings identified a strong negative correlation between diabetes mortality and IHDI (r = -0.884), which underscores how broader socioeconomic development is associated with improved management of chronic diseases like diabetes. Additionally, PCA analysis further demonstrated that adolescent health metrics distinctly segregate Caribbean nations from higher-income countries, highlighting persistent healthcare access gaps (Figures 5A, 5B). In contrast, men experienced significantly higher external mortality across nearly all countries, a pattern that likely reflects greater male exposure to occupational hazards, road traffic injuries, and interpersonal violence, which are causes commonly observed in global mortality trends [17].

Reproductive health disparities also emerged as a defining element of gendered health inequality in the Caribbean. Maternal mortality rates in the region (9.39 per 10,000 live births ± 5.22) were substantially higher than those in North America, and adolescent fertility rates (34.26 per 10,000 ± 19.49) also exceeded those observed in high-income countries (p < 0.001 and p = 0.05, respectively). These patterns echo previous findings linking structural inequity and gender-based health disparities to weaker reproductive health systems and limited access to prenatal care [18,19]. Our analysis further revealed a negative correlation between adolescent fertility and GDP per capita (r = -0.710), reinforcing prior evidence that economic hardship is closely tied to early childbearing and long-term reproductive risk [20,21]. These associations support the growing recognition that reproductive and chronic disease outcomes are not separate phenomena, but rather interlinked manifestations of broader gendered and socioeconomic inequities in health systems across the Caribbean [22].

Socioeconomic conditions such as high income inequality and food insecurity continue to shape these health outcomes. Despite having the highest GDP per capita in the region, the Bahamas remains one of the most unequal societies, as indicated by its IHDI and GINI coefficients. This contradiction helps explain its simultaneous burden of economic wealth and elevated obesity prevalence, which we identified as the highest in the Caribbean. These patterns suggest that economic growth alone is insufficient to reduce chronic disease risk without equity-focused health and social policies. While social conditions remain central, emerging evidence also points to biological contributors. Genetic studies have identified African ancestry-linked variants, such as ZRANB3, that impair insulin secretion and increase diabetes risk [23], reinforcing the critical role of the insulin secretory pathway, where defects can directly contribute to the development of diabetes.

Similar disparities have been observed across other chronic conditions. For instance, studies show that cataract burden in Caribbean small island developing states (SIDS) disproportionately affects socioeconomically disadvantaged populations [24]. Economic constraints often push families toward low-cost, ultra-processed foods, a pattern that has been extensively documented in food insecurity research, particularly in countries like Haiti [19,25]. These outcomes reinforce longstanding evidence that social and economic inequality, rather than individual behavior, is the dominant force shaping population health [19,26,27].

The Caribbean’s economic dependence on tourism further compounds these structural vulnerabilities. While tourism boosts national GDP, it often generates low-wage, seasonal employment with limited job security or healthcare benefits. This economic structure mirrors prior findings on how gender-based and economic vulnerabilities shape health outcomes in the Caribbean [22]. As a result, the apparent economic growth tied to tourism has not translated into improved or equitable health outcomes across the region. Recent studies of breast cancer and other chronic disease risks in the Caribbean show that economic growth has not translated into equitable health outcomes, reinforcing how persistent social and economic divides continue to widen regional health disparities [22]. These findings reinforce the need for economic diversification and gender-responsive health financing strategies that strengthen long-term health resilience.

Our PCA-derived clusters offer further insight into how structural inequality manifests across the region. Cuba’s clustering alongside high-income countries like the U.S. and Canada is striking, given its much lower GDP ($103.43 billion vs. $1.74 trillion for Canada and $21.54 trillion for the U.S. in 2019) and ongoing economic constraints [28]. Cuba’s strong public health indicators may reflect its decades-long investment in preventive care and universal health coverage, though concerns persist regarding the sustainability of these gains and the reliability of reported data amid deepening resource limitations [29]. These clustering patterns may also partially reflect broader geographic and economic distinctions. For instance, some higher-performing small island states, such as Barbados and Trinidad, clustered together, while more vulnerable Caribbean nations, including Haiti and Jamaica, grouped separately. However, not all clusters aligned neatly with geographic or linguistic categories, and we did not formally test such associations.

In contrast, countries in the red cluster, such as the Bahamas, Belize, Dominican Republic, Guyana, Haiti, Jamaica, Saint Vincent and the Grenadines, and Suriname, exhibited consistent patterns of socioeconomic vulnerability, under-resourced health systems, and disproportionately high gender-related disparities in NCD outcomes [4]. Meanwhile, countries in the green cluster, such as Antigua and Barbuda, Barbados, Grenada, Saint Lucia, and Trinidad and Tobago, tended to maintain stronger health metrics, likely supported by more stable governance structures and sustained public health investment. These findings highlight how structural stability and policy prioritization can buffer against broader regional inequities, while countries facing overlapping stressors may require more intensive, targeted interventions.

The wide gaps in policy implementation observed in this study underscore the urgent need for coordinated equity-driven public health reform across the Caribbean. While countries in North America have achieved broad adoption of foundational NCD prevention policies, most Caribbean countries remain at an early stage, particularly in the areas of child-targeted marketing, breastfeeding protections, and physical activity promotion [30]. These findings align with prior work showing that NCD risk in Caribbean youth is rising rapidly in the absence of structural policy safeguards, especially for obesity and diet-related conditions [7].

The complete absence of marketing restrictions to children across all 13 Caribbean countries included in this analysis is especially concerning given the region’s documented challenges with ultra-processed food exposure, school-based advertising, and limited regulatory enforcement [30]. Similarly, breastmilk substitute regulations, a critical tool for protecting infant nutrition, were implemented in only one country, reflecting a neglected policy space despite long-standing WHO recommendations. These gaps are not only technical oversights but manifestations of deeper systemic inequities that leave Caribbean women and children particularly vulnerable to preventable NCDs [4,9]. Several structural constraints likely contribute to this pattern. Past studies have highlighted barriers, including inconsistent health financing, limited legislative infrastructure, and poor integration of NCD services into primary care systems, particularly following environmental and economic shocks [3]. For small island states, the capacity to draft, enforce, and monitor public health legislation often remains limited, further reinforcing dependence on donor priorities and regional agencies for guidance and technical support.

Improving policy adoption will require more than regional consensus statements; it will demand clear political commitment, cross-country collaboration, and investment in health system readiness. Models like the PAHO ENLACE scorecard can provide useful benchmarking, but implementation support must go beyond measurement. Prior research has shown that achieving population-level impact requires aligning fiscal policies, regulatory tools, and public education strategies, particularly when tackling upstream drivers of NCDs such as poverty, food insecurity, and gender inequity [18].

Efforts to advance universal healthcare coverage, reduce out-of-pocket costs, and embed preventive services into routine care are particularly promising for women’s health outcomes in vulnerable settings [30]. Strengthening regional surveillance systems and enabling shared policy templates across ministries of health could also lower the administrative burden of reform. Ultimately, closing the implementation gap will require structural investment in public health governance that centers equity and resilience, ensuring that NCD prevention is not only planned, but enacted.

This study has several limitations. Its cross-sectional design limits causal inference, allowing only for the identification of associations between economic conditions, healthcare access, and health outcomes. The analysis relied on publicly available datasets from PAHO, UNDP, and IDF due to the limited availability of peer-reviewed data on Caribbean health metrics, which may introduce inconsistencies in data quality across countries. Data imputation was used sparingly and only for the Pearson correlation and PCA analyses to preserve sample size; while necessary, this may have introduced minor bias. Additionally, data collection disruptions during the COVID-19 pandemic limited the availability of recent socioeconomic indicators, particularly for smaller Caribbean states. As an ecological study, these findings reflect country-level associations and cannot be extrapolated to individual-level patterns, introducing a risk of ecological fallacy. Furthermore, variation in reporting practices across international sources may have introduced additional inconsistencies despite efforts to harmonize data.

## Conclusions

This study concludes that women in the non-Latin Caribbean region face distinct health risks, including higher obesity and diabetes-related mortality compared to men. Gender gaps were also evident in reproductive health, with elevated maternal mortality and adolescent fertility rates in several countries. Although not uniform across the region, principal component analysis showed that nations with weaker reproductive health outcomes clustered together, reflecting shared vulnerabilities. Compared to North America, the Caribbean overall demonstrated a higher chronic disease burden, poorer reproductive health indicators, and lower development scores, underscoring the need for region-specific public health strategies.

These findings carry important implications for policy and planning. Leaders must prioritize strategies that address chronic and reproductive health risks among women, with an emphasis on prevention, access, and equity. Expanding maternal health services, reducing adolescent fertility through comprehensive education, and strengthening chronic disease prevention are immediate priorities. Policy gaps identified through the ENLACE tool, particularly in food marketing to children and breastfeeding protections, represent clear opportunities for action. Regional collaboration can support shared policy frameworks and more consistent implementation across countries. By offering one of the most comprehensive cross-country analyses on Caribbean women, this study provides a data-driven foundation for advancing gender-responsive health systems.

## Appendices

Data availability statement

This study utilized publicly available data from multiple sources; we can provide all obtained data upon request. Health indicators, including noncommunicable disease (NCD) prevalence, diabetes mortality rates, and other health metrics, were obtained from the Pan American Health Organization (PAHO) Core Indicators Portal (https://opendata.paho.org/en/core-indicators/core-indicators-dashboard). Socioeconomic metrics, including the Inequality-Adjusted Human Development Index (IHDI), unemployment rates, and national development indicators, were sourced from the United Nations Development Programme (UNDP) Human Development Reports (https://hdr.undp.org/data-center/human-development-index#/indicies/HDI) and the World Bank World Development Indicators (https://data.worldbank.org/indicator). Diabetes-related data, including gestational diabetes prevalence and regional burden estimates, were retrieved from the International Diabetes Federation (IDF) Diabetes Atlas 2021 (https://diabetesatlas.org/data/en/). Geographic shapefiles used for regional mapping were obtained from the Database of Global Administrative Areas (GADM, https://gadm.org/data.html), and maps were generated in QGIS (https://qgis.org). U.S. diabetes prevalence estimates were drawn from the Centers for Disease Control and Prevention (CDC). All datasets are publicly accessible, and further details on data retrieval can be provided upon request.

**Table 1:** Master table of variables for Non-latin Caribbean nations, Canada, and The United States Variables compiled from the Pan American Health Organization (PAHO), United Nations Development Programme (UNDP), and International Diabetes Federation (IDF), with country-level coverage indicated. Countries without a metric for a given variabel are denoted as 'NA' for Not Available.

Country	External causes mortality rate (ageadjusted per 100 000 pop); male in 2019	External causes mortality rate (ageadjusted per 100 000 pop); female in 2019	Deaths from noncommunicable diseases (%); male in 2019	Deaths from noncommunicable diseases (%); female in 2019	Deaths from communicable diseases (%); male in 2019	Deaths from communicable diseases (%); female in 2019	Prevalence of raised blood pressure in adults (%); male in 2019	Diabetes mellitus mortality rate (ageadjusted per 100 000 pop); male in 2019	Diabetes mellitus mortality rate (ageadjusted per 100 000 pop); female in 2019	General mortality rate (ageadjusted per 1 000 pop); male in 2019	General mortality rate (ageadjusted per 1 000 pop); female in 2019	Proprotion men	Proportion women	Diabetes mellitus mortality rate (ageadjusted per 100 000 pop) in 2019	Infant mortality rate (1 000 lb) in 2019	Maternal mortality ratio (100 000 lb) in 2019	Adolescent fertility rate (births per 1 000 women aged 1519 years) in 2019	Life expectancy at birth (years); female in 2019	Median age (years) in 2019	Gross domestic product (US$ per capita), current international (PPPadjusted) in 2019	Unemployment rate (%); female in 2019	Prevalence of moderate or severe food insecurity in the population (%) in 2019	2019 HDI	2019 IHDI	Inflation (%)	Unemployment rate (%); male in 2019	Urban Population (%) in 2019	Suicide mortality rate (ageadjusted per 100 000 pop); female IN 2019	Ischemic heart diseases mortality rate (ageadjusted per 100 000 pop); male in 2019	Ischemic heart diseases mortality rate (ageadjusted per 100 000 pop); female in 2019	Stroke diseases mortality rate (ageadjusted per 100 000 pop); male in 2019	Stroke diseases mortality rate (ageadjusted per 100 000 pop); female in 2019	Respiratory diseases mortality rate (ageadjusted per 100 000 pop); male in 2019	Respiratory diseases mortality rate (ageadjusted per 100 000 pop); female in 2019	Cirrhosis mortality rate (ageadjusted per 100 000 pop); male in 2019	Cirrhosis mortality rate (ageadjusted per 100 000 pop); female in 2019	Stomach cancer mortality rate (ageadjusted per 100 000 pop); male in 2019	Stomach cancer mortality rate (ageadjusted per 100 000 pop); female in 2019	Prevalence of overweight and obesity in adults (%); male in 2019	Prevalence of overweight and obesity in adults (%); female in 2019	Prevalence of insufficient physical activity in adults (%); male in 2019	Prevalence of insufficient physical activity in adults (%); female in 2019	Outofpocket expenditure as % of current health expenditure in 2019	UHC index in 2019 WHO GHO	Adolescent mortality rate (per 1 000 age specific cohort) 1519 female WHO GHO	Low birth weight prevalence (%) IN 2019 who gho	Current health expenditure (CHE) as percentage of gross domestic product (GDP) (%) in 2019 WHO GHO	Preterm birth rate (per 100 live births) IN 2019 who gho	Prevalence of raised blood pressure in adults (%)	Homicide mortality rate (age-adjusted per 100 000 pop); female in 2019	IDF Hyperglyceia in Pregnancy in 2021
Anguilla	NA	NA	NA	NA	NA	NA	NA	NA	NA	NA	NA	NA	NA	NA	15.4	NA	29.7	82.1	35.5	NA	NA	NA	NA	NA	NA	NA	100	NA	NA	NA	NA	NA	NA	NA	NA	NA	NA	NA	NA	NA	NA	NA	NA	NA	1.5	NA	NA	NA	NA	NA	NA
Antigua and Barbuda	55.3	15.4	81.9	87.9	10.4	9.2	34.4	56	66.1	6.7	5.3	8.36	12.47	61.5	NA	NA	31.7	79.8	33.8	26317	NA	33	0.83	NA	1.4	NA	24.5	0.6	89.5	67.2	63.4	68.3	31.3	19	5.1	1.2	9.4	4.3	52.5	68.5	29.8	43.3	24.3	74.57	1.28	4.41	4.41	NA	34.4	1.2	NA
Aruba	NA	NA	NA	NA	NA	NA	NA	NA	NA	NA	NA	NA	NA	NA	1	NA	22.1	78.8	40.1	40125	NA	0	NA	NA	4.3	NA	43.5	NA	NA	NA	NA	NA	NA	NA	NA	NA	NA	NA	NA	NA	NA	NA	NA	NA	NA	NA	NA	NA	NA	NA	NA
Bahamas	149.2	39.3	70.3	82.3	10.7	10.6	37.8	53.4	48.2	8.7	5.5	6.14	8.76	50.6	19.4	118.5	26	74.2	33.2	34542	10	NA	0.8	0.64	2.5	10.1	83.1	1.2	117.9	65.1	67.4	54.2	20.4	9.5	23.6	9.8	7	2.9	68.8	79.1	26.9	46.5	26.7	78.75	4.65	5.87	5.87	NA	37.8	9	NA
Barbados	52.4	22.1	81.9	83.6	12.6	13.1	28.4	43.4	47.6	6.8	5.3	6.38	8.98	45.9	8.6	78.1	46	78.5	37.9	17459	7.3	31.1	0.81	0.61	4.1	9.5	31.2	0.2	65.6	46.7	63	54.2	26	13.2	9.1	1.4	7.2	3.2	56.9	74	35.1	49.9	46.7	78.7	1.4	6.12	6.12	NA	28.4	4.6	NA
Belize	166.1	32.3	59.4	74.5	14.8	17.8	32	62.1	71.2	7.8	5.1	7.96	13.96	66.5	15.3	123.8	65.6	75.7	24.3	10436	13.9	45.5	0.72	NA	0.2	6	45.9	1.8	87.5	59.1	53.5	43.2	36.1	14.6	27.5	13	11.4	5.6	64.1	78.3	34.1	46.5	23.1	69.23	3.19	4.84	4.84	NA	32	10.3	NA
Bermuda	NA	NA	NA	NA	NA	NA	NA	NA	NA	NA	NA	NA	NA	NA	1.9	NA	3	85	43.7	91263	NA	0	NA	NA	NA	NA	100	NA	NA	NA	NA	NA	NA	NA	NA	NA	NA	NA	52.5	68.6	NA	NA	NA	NA	NA	NA	NA	NA	NA	NA	NA
Cayman Islands	NA	NA	NA	NA	NA	NA	NA	NA	NA	NA	NA	NA	NA	NA	6.1	NA	11.5	81.6	36.4	74009	NA	0	NA	NA	NA	NA	100	NA	NA	NA	NA	NA	NA	NA	NA	NA	NA	NA	NA	NA	NA	NA	NA	NA	NA	NA	NA	NA	8.8	NA	NA
Cuba	62.9	28.3	82.8	83.9	9	9.5	29.2	11.1	13	6.4	4.2	1.73	3.1	12.2	5	37.4	51.1	79.9	41.1	9232	1.2	0	0.77	NA	NA	1.2	77.1	4.1	118.1	79	54.7	40.9	33.7	23.9	17.7	3.4	6.4	3.5	50.3	55.7	46.6	68.2	10.9	83.41	1.5	11.09	11.09	5.97	19.6	2.3	NA
Curaçao	NA	NA	NA	NA	NA	NA	NA	NA	NA	NA	NA	NA	NA	NA	5.7	NA	15.8	80.6	36.6	25397	NA	NA	NA	NA	2.6	NA	89.1	NA	NA	NA	NA	NA	NA	NA	NA	NA	NA	NA	NA	NA	NA	NA	NA	NA	NA	NA	NA	NA	29.2	NA	NA
Dominica	NA	NA	NA	NA	NA	NA	39	NA	NA	NA	NA	NA	NA	NA	22.1	NA	36.2	74.5	34	14452	NA	34.4	0.75	NA	1.5	NA	70.8	NA	NA	NA	NA	NA	NA	NA	NA	NA	NA	NA	40.7	73.6	19.9	37.5	33.9	71.93	1.06	5.17	5.17	NA	NA	NA	NA
Dominican Republic	182.8	36.4	66.2	79.6	11.6	13.9	40.2	36.2	31.8	8.5	5.6	4.26	5.68	34	20.6	90.7	67	76.4	26.4	19743	9.7	52.1	0.77	0.61	1.8	4.1	81.8	1.9	174.7	116.4	86.1	68.2	21.5	15.1	34.4	19.2	6.9	4.3	55.1	66.7	29.9	41.3	27.9	75.05	2.5	4.24	4.24	NA	39	5	NA
French Guiana	NA	NA	NA	NA	NA	NA	NA	NA	NA	NA	NA	NA	NA	NA	8.2	NA	66.8	79.9	25	NA	NA	NA	NA	NA	NA	NA	85.6	NA	NA	NA	NA	NA	NA	NA	NA	NA	NA	NA	NA	NA	NA	NA	NA	NA	NA	NA	NA	NA	23	NA	NA
Grenada	74.6	22.1	81.7	84.2	10.3	12.6	40.6	87.4	74.8	9	6.3	9.71	11.87	80.5	10.3	63.6	31.6	78.2	31.4	15542	NA	21.1	0.79	NA	0.6	NA	36.4	0.7	136.7	94.7	80.3	67.4	25.2	14.4	13.8	2.7	7.9	4.6	47.8	68.6	27.6	41.3	54.4	73.83	1.04	4.93	4.93	NA	NA	2.2	NA
Guadeloupe	NA	NA	NA	NA	NA	NA	NA	NA	NA	NA	NA	NA	NA	NA	7.5	NA	9.6	85.1	43.8	NA	NA	NA	NA	NA	NA	NA	98.5	NA	NA	NA	NA	NA	NA	NA	NA	NA	NA	NA	NA	NA	NA	NA	NA	NA	NA	NA	NA	NA	40.6	NA	NA
Guyana	218.8	53.3	64.4	76.7	17.5	17.3	31.9	93.9	99.1	13.4	9.2	7.01	10.77	96.8	23	174.9	75.6	72.8	24.6	13389	15.1	25.5	0.71	NA	2.1	12.5	26.7	17	228.9	157.6	154.5	142.8	54.1	26.6	41.2	13.4	5.2	4.7	43.9	64.3	23.3	43.3	35	77.41	3.72	4.9	4.9	11.55	26.8	10.2	NA
Haiti	141.8	56.8	59.4	69.7	25.3	24	34.7	52.7	102.6	11.9	11.2	4.43	9.16	79.9	NA	NA	53.6	67.3	22.5	3221	17.1	82.6	0.56	0.34	18.7	11.4	56.2	8	190.3	180.4	136.9	183.6	61.6	41.3	41.2	22	15.9	8.5	21	36.5	19.6	34.3	39	52.79	5.62	3.91	3.91	NA	31.9	8.1	NA
Jamaica	112	35.1	73.3	86.5	10.5	7.1	38.3	61.2	78.5	6.6	5.1	9.27	15.39	70.1	18.4	163.5	37.7	74	29.7	10118	6.4	54.4	0.71	0.59	3.9	3.8	56	1	54.3	45.4	68	72.3	25.3	7.9	6.2	3.5	8.7	5.4	45.2	72.4	30.1	44	16.4	76.58	2.53	6.1	6.1	NA	24.7	20.4	NA
Martinique	NA	NA	NA	NA	NA	NA	NA	NA	NA	NA	NA	NA	NA	NA	7.2	NA	11.3	85.1	47	NA	NA	NA	NA	NA	NA	NA	89.1	NA	NA	NA	NA	NA	NA	NA	NA	NA	NA	NA	NA	NA	NA	NA	NA	NA	NA	NA	NA	NA	38.3	NA	NA
Montserrat	NA	NA	NA	NA	NA	NA	NA	NA	NA	NA	NA	NA	NA	NA	NA	NA	17.8	78.2	40.3	NA	NA	NA	NA	NA	NA	NA	9.1	NA	NA	NA	NA	NA	NA	NA	NA	NA	NA	NA	NA	NA	NA	NA	NA	NA	1.51	NA	NA	NA	25.8	NA	NA
Puerto Rico	NA	NA	NA	NA	NA	NA	34.3	NA	NA	NA	NA	NA	NA	NA	6.6	34.4	18.9	85.1	43.7	37454	6.6	0	NA	NA	NA	9.5	93.6	NA	NA	NA	NA	NA	NA	NA	NA	NA	NA	NA	70.2	73.8	35.8	44.7	NA	NA	NA	NA	NA	NA	19.6	NA	NA
Saint Kitts and Nevis	NA	NA	NA	NA	NA	NA	37.5	NA	NA	NA	NA	NA	NA	NA	16.8	168.4	39.6	75.5	33.7	31965	NA	NA	0.84	NA	0.3	NA	30.8	NA	NA	NA	NA	NA	NA	NA	NA	NA	NA	NA	67.2	80.3	33	47.3	46.4	76.78	3.53	5.19	5.19	NA	34.3	NA	NA
Saint Lucia	118.8	27.8	79.2	86.7	7.5	8.4	34.1	74.4	67.2	8.1	5	9.19	13.44	70.6	15.2	52.5	30.8	76.1	32.2	18584	17.3	NA	0.73	0.55	0.5	14.1	18.8	1.5	76.7	44.7	86.5	67.6	43.8	14.3	21.2	10.3	14	5.2	47.6	73.8	17.6	24.8	42.9	76.52	2.21	4.17	4.17	NA	37.5	6.9	NA
Saint Vincent and the Grenadines	100.5	23.8	75.3	83.6	13.1	13.1	31.5	44	61.3	7.8	6.1	5.64	10.05	52.9	10.8	NA	53.2	74	32.7	15234	17	NA	0.79	NA	0.9	20.7	52.6	0.7	99.4	87.4	75.5	70.2	42.1	27.1	7.3	2	9.9	4.4	46.9	73.3	20.7	38.6	28.1	76.3	3.14	4.26	4.26	NA	34.1	11.8	NA
Sint Maarten (Dutch part)	NA	NA	NA	NA	NA	NA	NA	NA	NA	NA	NA	NA	NA	NA	NA	NA	19.5	78.6	39.6	46565	NA	0	NA	NA	NA	NA	100	NA	NA	NA	NA	NA	NA	NA	NA	NA	NA	NA	NA	NA	NA	NA	NA	NA	NA	NA	NA	NA	31.5	NA	NA
Suriname	114.8	40.4	75.2	83	12.4	11	34.7	83.9	62.8	10.6	6.6	7.92	9.52	72	NA	89.1	53.2	75.1	27.3	19952	10.6	35.8	0.71	NA	NA	5.4	66.1	11.8	164.6	90.2	138.4	100.7	34.2	17.3	35.3	15.1	6.8	2.8	47.2	65.2	40.7	53.2	17.4	72.3	4.24	8.53	8.53	12.84	NA	2.7	NA
Trinidad and Tobago	116.7	21	78.9	88	6.3	8	35.7	95.4	63.1	7.2	4.2	13.25	15.02	77.6	9.6	25.7	38	76	34.7	26641	4	43.3	0.81	NA	1	3.1	53.2	3.7	109.3	58.9	56	37.7	16.7	6.7	11.6	4.3	5.6	2.3	52.6	61.3	33.7	52.7	46.9	76.02	2.77	6.85	6.85	NA	34.7	7.7	NA
Turks and Caicos Islands	NA	NA	NA	NA	NA	NA	NA	NA	NA	NA	NA	NA	NA	NA	NA	NA	19.1	80	36.7	26806	NA	0	NA	NA	NA	NA	93.4	NA	NA	NA	NA	NA	NA	NA	NA	NA	NA	NA	NA	NA	NA	NA	NA	NA	1.6	NA	NA	NA	35.7	NA	NA
Virgin Islands (British)	NA	NA	NA	NA	NA	NA	NA	NA	NA	NA	NA	NA	NA	NA	NA	NA	13.5	79.6	35.9	NA	NA	NA	NA	NA	NA	NA	48.1	NA	NA	NA	NA	NA	NA	NA	NA	NA	NA	NA	NA	NA	NA	NA	NA	NA	NA	NA	NA	NA	34.5	NA	NA
Virgin Islands (U.S.)	NA	NA	NA	NA	NA	NA	NA	NA	NA	NA	NA	NA	NA	NA	NA	NA	32.2	80.9	44.1	40022	13.2	0	NA	#N/A	NA	11.5	95.8	NA	NA	NA	NA	NA	NA	NA	NA	NA	NA	NA	NA	NA	NA	NA	NA	NA	NA	NA	NA	NA	29.7	NA	NA
Canada	37.3	16.6	89	90.6	4.5	5.1	8.8	11.1	6.2	4.1	2.9	2.71	2.14	8.5	4.4	8	6.4	84.4	39.8	50499	5.3	7.7	0.93	0.86	1.9	6.1	81.5	5.4	61.4	30.6	15.9	14.3	26.5	19.3	7.7	3.8	3.9	2	64.8	53.2	34	37.2	15.5	90.53	1.24	11.06	11.06	7.92	NA	0.8	NA
United States	66.9	25.4	86.3	90.1	5.1	5.5	18.8	18.5	11.5	5.7	4	3.25	2.88	14.8	5.6	20.1	16.2	81.5	37	65548	3.6	8.6	0.93	0.82	1.8	3.7	82.5	6.8	97.8	52.5	22.8	21.4	44	35.5	11.7	6.4	2.9	1.7	74.1	68.6	27.3	39.1	11.3	85	1.5	8.2	16.66	10	NA	2.3	NA
